# Supplementary figures and images for: Thymidylate synthase promotes esophageal squamous cell carcinoma growth by relieving oxidative stress through activating nuclear factor erythroid 2-related factor 2 expression
Source: PLoS One. 2023 Sep 8;18(9):e0290264. doi: 10.1371/journal.pone.0290264 (PMC10490860; doi:10.1371/journal.pone.0290264)

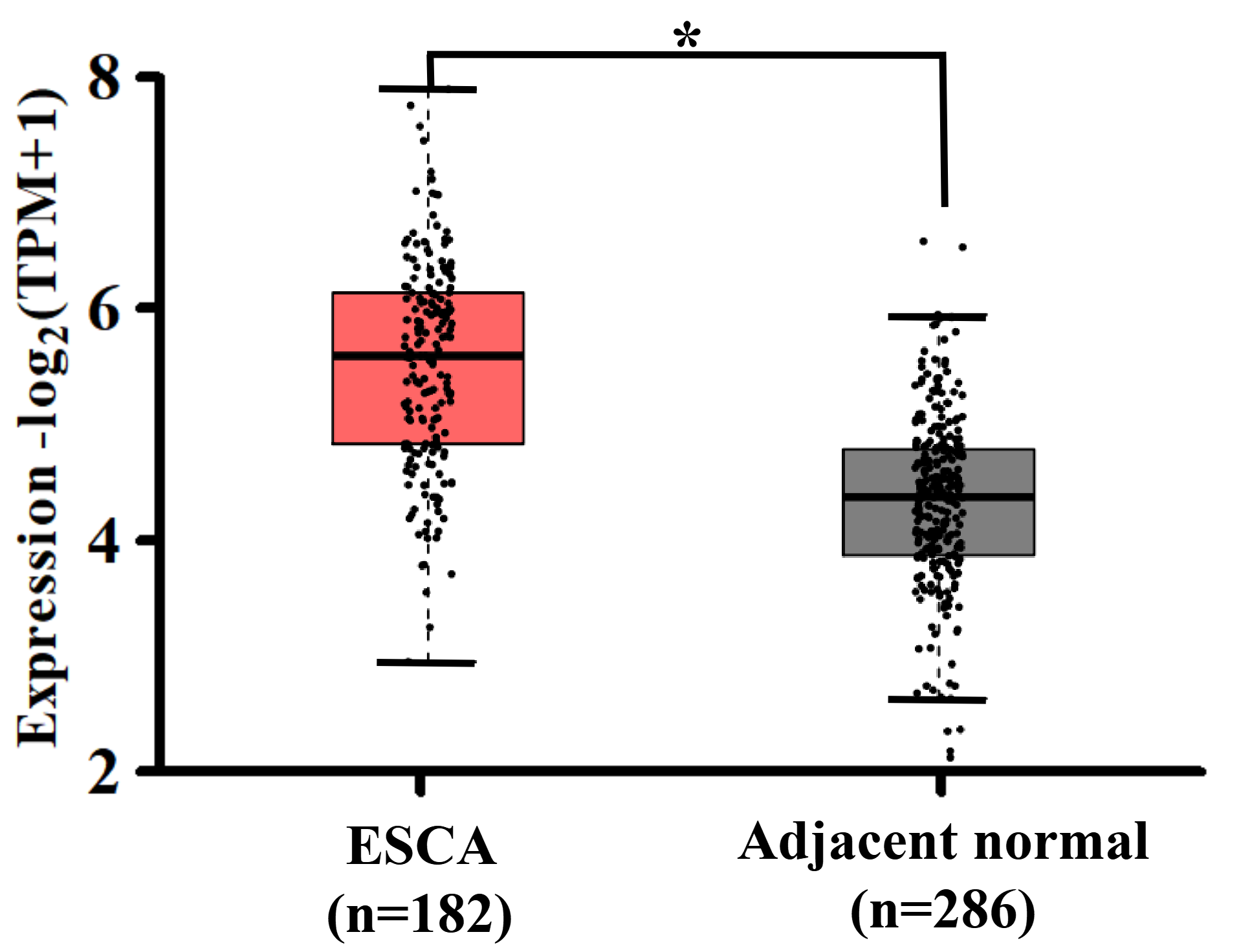

Supplement: S1 Fig — (TIF) [file pone.0290264.s001.tif]

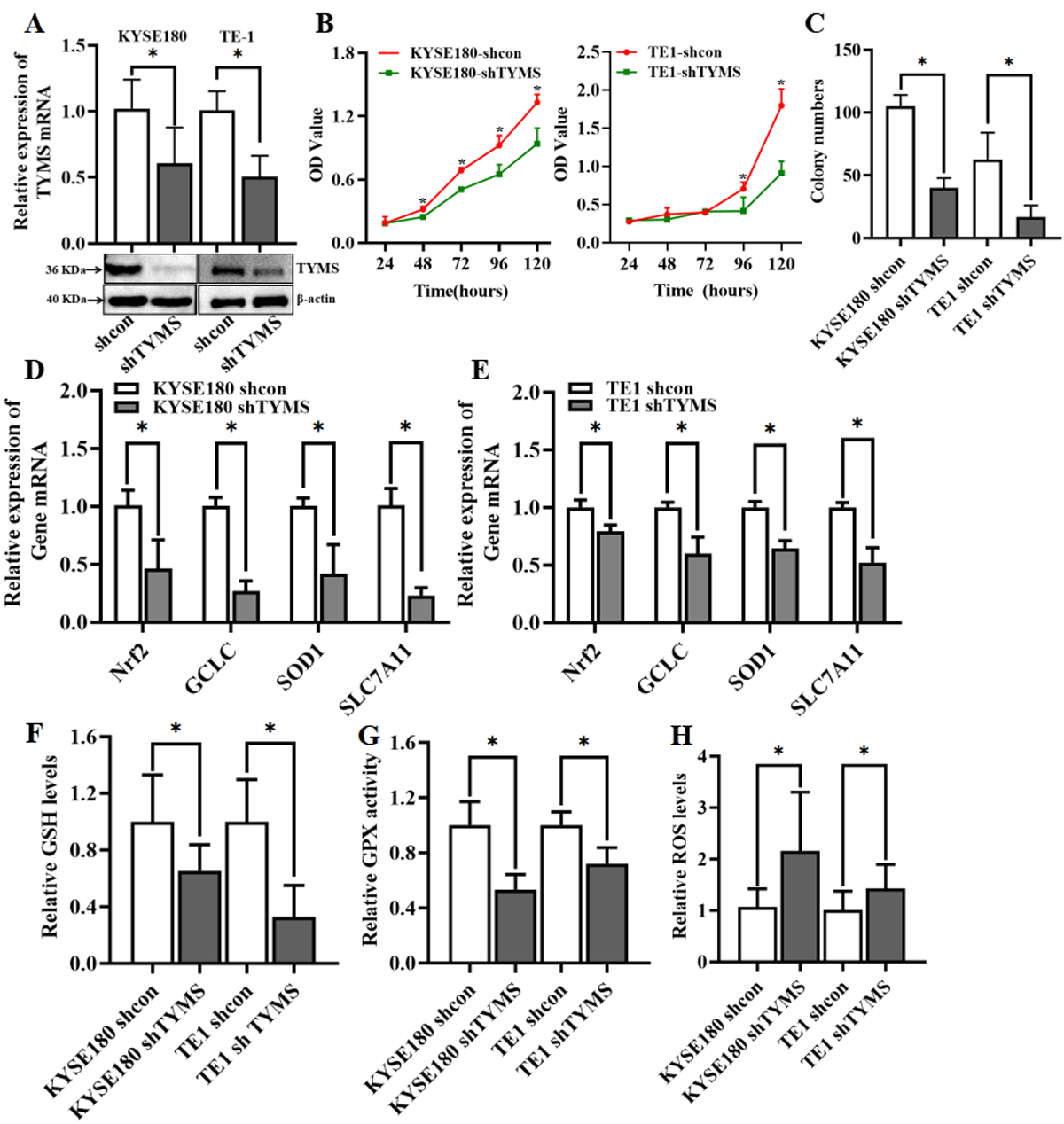

Supplement: S2 Fig — (A) Knockdown efficiency of TYMS in KYSE180 and TE-1 cells was detected through qRT-PCR and Western blot. MTT (B) and clonal formation experiment (C) detected the cell proliferation ability after knockdown of TYMS in KYSE180 and TE-1. (D and E) qRT-PCR detected the expression of Nrf2 and Nrf2 dependent antioxidant enzyme genes in TYMS-knockdown cells. GSH levels (F), GPX activity (G), and ROS levels (H) in TYMS-knockdown cells and correspondence control cells. Data were representative of least three independent experiments and were presented as mean ± S.D., where a p value < 0.05 was regarded as statistically significant. (TIF) [file pone.0290264.s002.tif]
