# Supplementary material for: Thymidylate synthase promotes esophageal squamous cell carcinoma growth by relieving oxidative stress through activating nuclear factor erythroid 2-related factor 2 expression
Source: PLoS One. 2023 Sep 8;18(9):e0290264. doi: 10.1371/journal.pone.0290264 (PMC10490860; doi:10.1371/journal.pone.0290264)

Figure 1A： TYMS

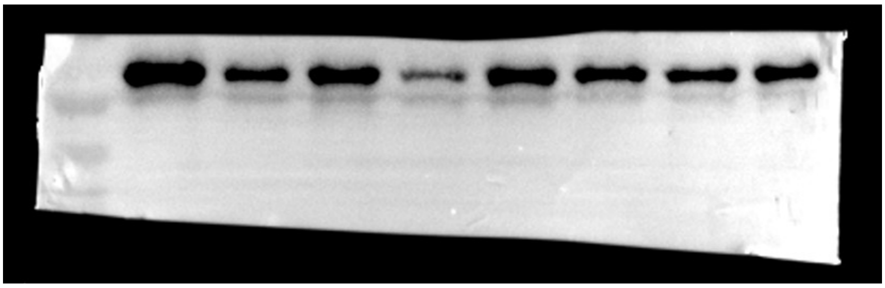

Figure 1A： β-actin

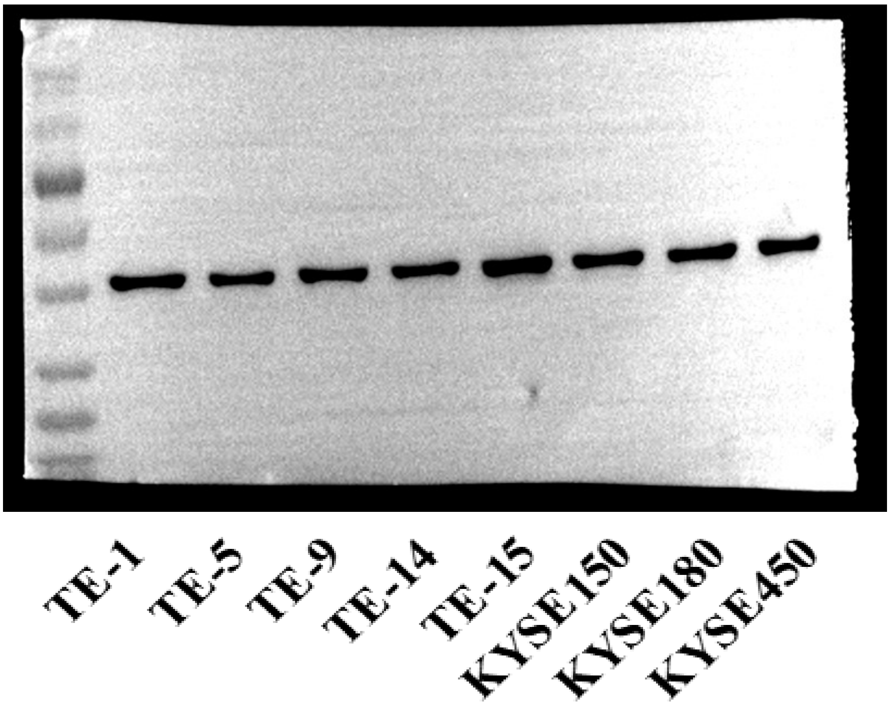

Figure 1B： KYSE150 TYMS

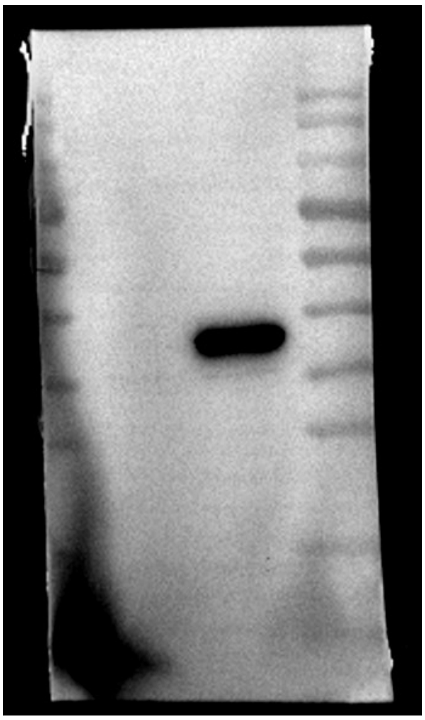

Figure 1B： KYSE150 β-actin

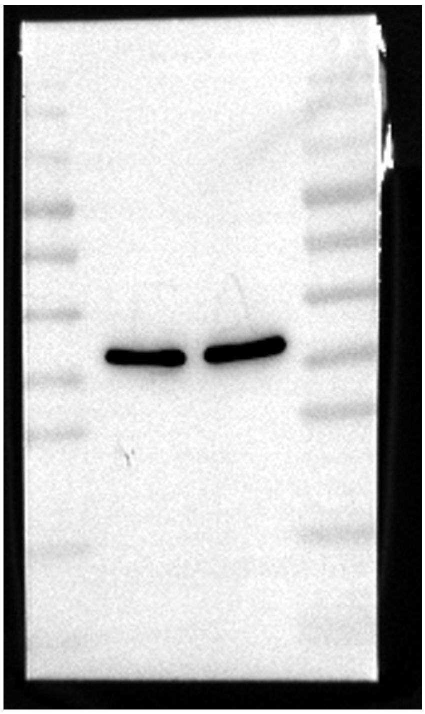

Figure 1B：
KYSE180 TYMS

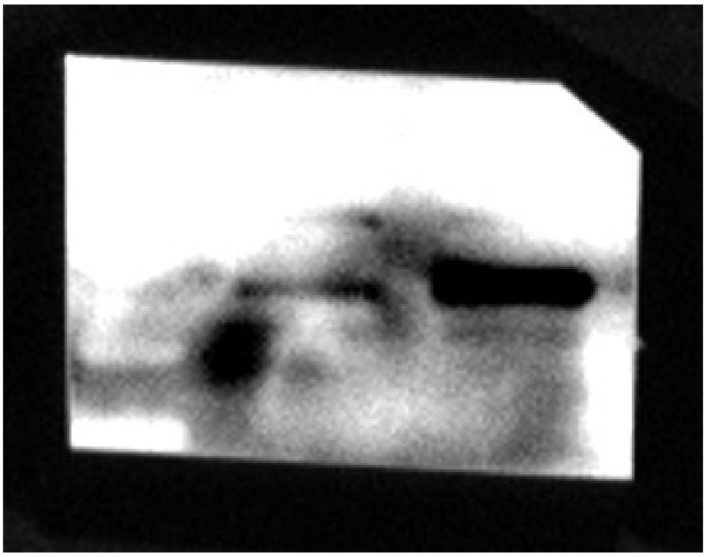

NC TYMS

Figure 1B：
KYSE180 β-actin

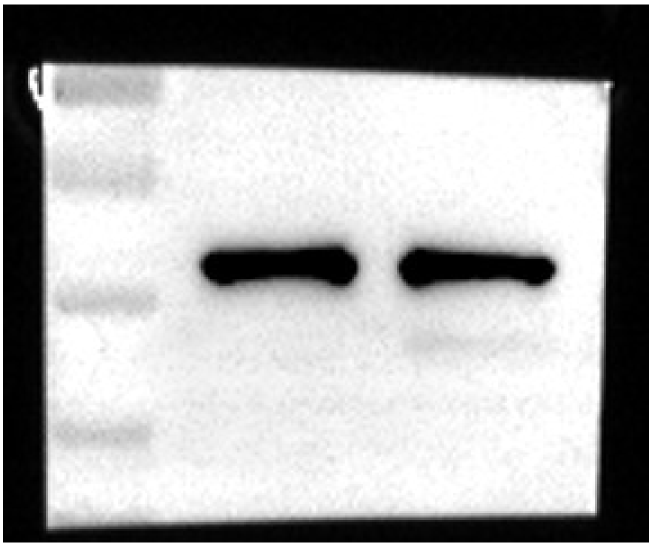

NC TYMS

S2 FigA： KYSE180 TYMS

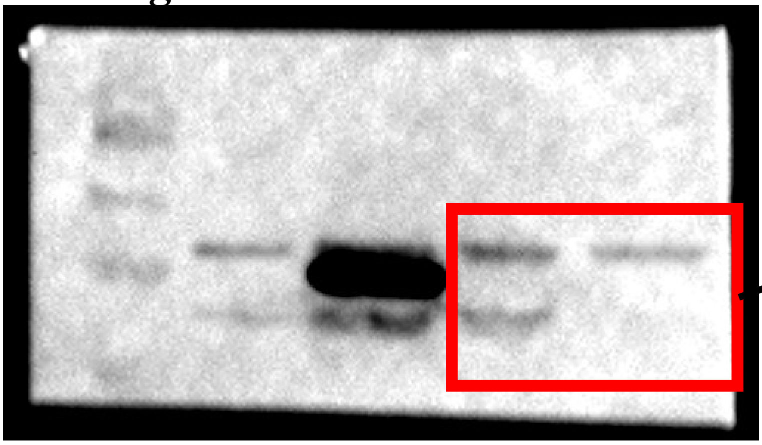

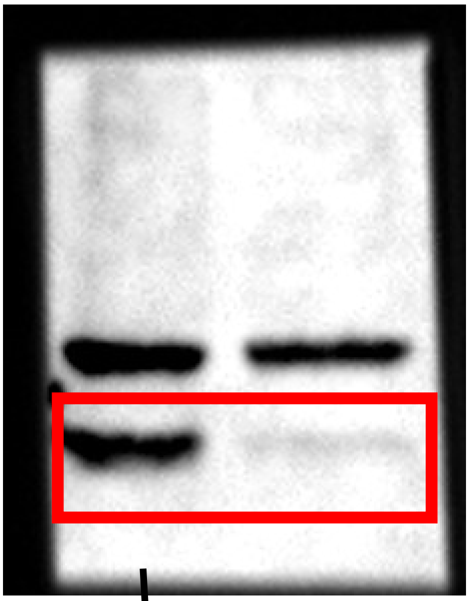

TYMS

S2 FigA： KYSE180 β-actin

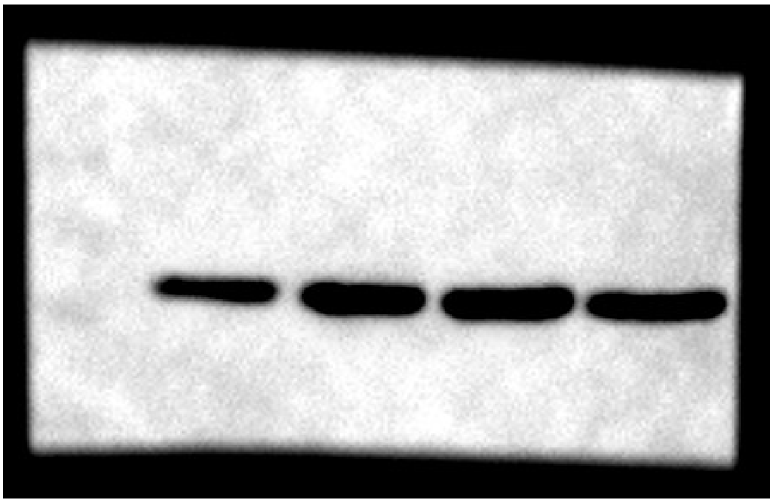

NC TYMS shcon shTYMS

S2 FigA： TE-1 TYMS

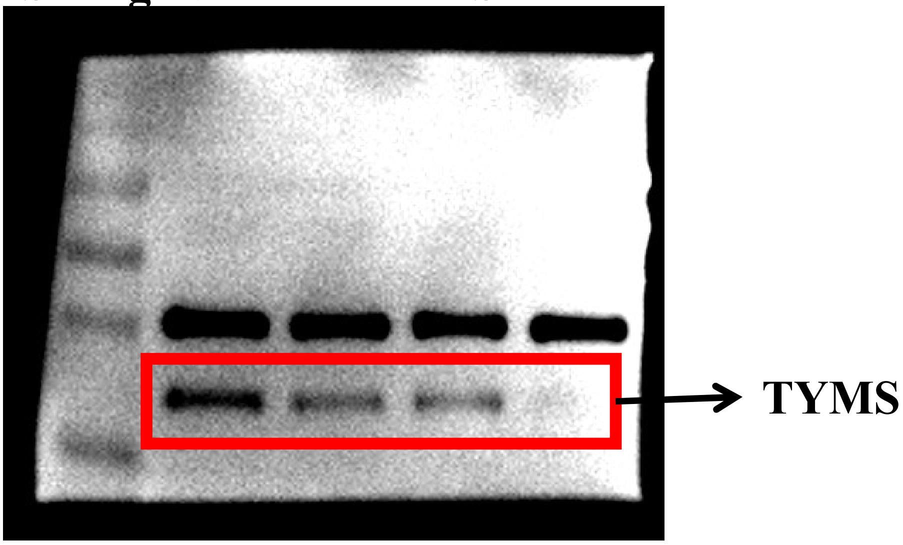

S2 FigA： TE-1 β-actin

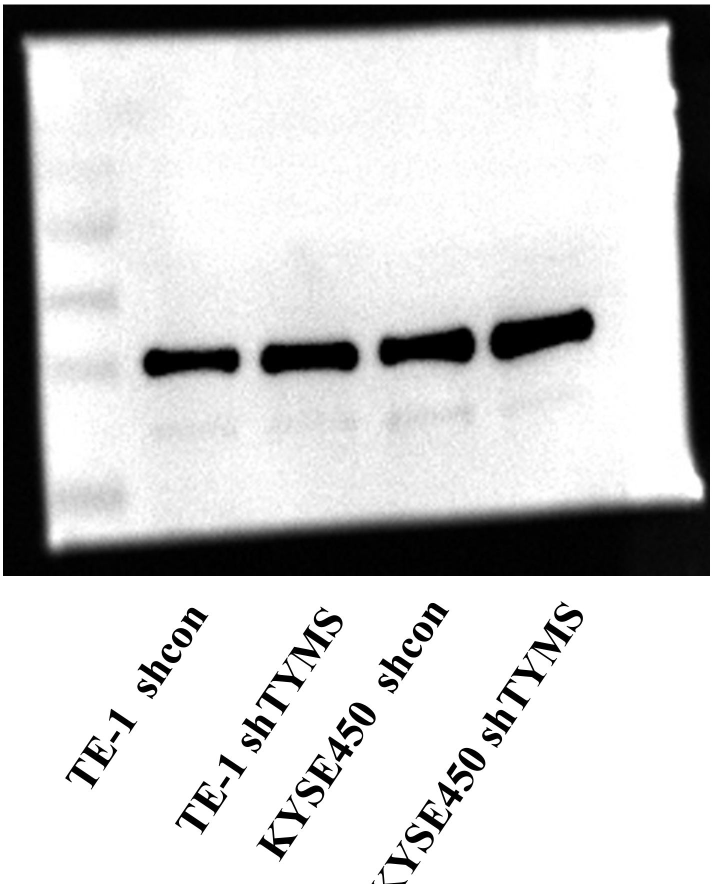

Supplement: S1 Raw images — (PDF) [file pone.0290264.s003.pdf]
