# Supplementary material for: Thymidylate synthase promotes esophageal squamous cell carcinoma growth by relieving oxidative stress through activating nuclear factor erythroid 2-related factor 2 expression
Source: PLoS One. 2023 Sep 8;18(9):e0290264. doi: 10.1371/journal.pone.0290264 (PMC10490860; doi:10.1371/journal.pone.0290264)
Supplement: Attachment — Submitted filename: Responses to Reviewers.doc [file pone.0290264.s004.doc]

**Responses to Reviewers**

Dear Editor

We want to thank you for your useful comments and suggestions on our manuscript. We have revised the manuscript accordingly, and detailed corrections are listed below:

**Comments of Editor:**

Two experts read your manuscript thoroughly and evaluated the merit for publication in PLOS ONE. As you can find their constructive comments below, they found your work interesting but also noticed several important issues and concerns to be clarified or addressed before further consideration of your work for publication in PLOS ONE. I see that the reviewers are in general agreement with their major concerns, such as the lack of appropriate gene expression analyses of TYMS and Nrf2 along with TYMS knockdown approaches, the lack of the molecular mechanism through which TYMS upregulates Nrf2, and author's interpretation not fully supported by the data. In addition, both reviewers have more specific comments and critiques on almost all figures. I believe that all comments and critiques from the reviewers are critical and important for improvement of your manuscript. You will need to set up new experiments to address their comments. Below you can find reviewer's specific comments and suggestions to improve your manuscript.

Author Response:

Thanks a lot for your suggestion and comments. Your suggestion is very necessary for the present study to clear the mechanism of overexpressed TYMS on Nrf2. Before submission, we tried to establish a TYMS stable knockout cell lines in TE-1 or other cell lines using Lentivirus Vector. However, it was hard to construct TYMS-silencing cells, and we fail until the paper submitted to the journal. Fortunately, we finally succeed to construct TYMS-silencing cells during the review period, and also added relevant experiments (Cell proliferation assays, Gene expression analysis, Detection of GSH, ROS, and GPx) in TYMS-silencing cells. The related results could be found in S1 Figure. The Nrf2-knockdown experiments were also difficult. ML385, an NRF-2 inhibitor, was used to replace the Nrf2-knockdown experiments, and confirmed that ML385 treatment in TYMS-overexpression cells re-increased the oxidative stress levels and the slowing-down cancer cell proliferation. Moreover, the comments and critiques from the reviewers are critical and important on the present manuscript, and we are very grateful for their generous help. According to the reviewer's suggestions, we carefully revised the manuscript, and also response to their questions point by point. Thanks again.

**Journal Requirements:**

When submitting your revision, we need you to address these additional requirements.

1). Please ensure that your manuscript meets PLOS ONE's style requirements, including those for file naming. The PLOS ONE style templates can be found at <https://journals.plos.org/plosone/s/file?id=wjVg/PLOSOne_formatting_sample_main_body.pdf> and https://journals.plos.org/plosone/s/file?id=ba62/PLOSOne_formatting_sample_title_authors_affiliations.pdf

Author Response:

Thank you for your suggestions, and modifications including format among others of Abstract, Introduction, Materials and Methods have been made according to PLOS ONE's style requirements.

2). PLOS ONE now requires that authors provide the original uncropped and unadjusted images underlying all blot or gel results reported in a submission’s figures or Supporting Information files. This policy and the journal’s other requirements for blot/gel reporting and figure preparation are described in detail at https://journals.plos.org/plosone/s/figures#loc-blot-and-gel-reporting-requirements and https://journals.plos.org/plosone/s/figures#loc-preparing-figures-from-image-files. When you submit your revised manuscript, please ensure that your figures adhere fully to these guidelines and provide the original underlying images for all blot or gel data reported in your submission. See the following link for instructions on providing the original image data: https://journals.plos.org/plosone/s/figures#loc-original-images-for-blots-and-gels.

In your cover letter, please note whether your blot/gel image data are in Supporting Information or posted at a public data repository, provide the repository URL if relevant, and provide specific details as to which raw blot/gel images, if any, are not available. Email us at plosone@plos.org if you have any questions.

Author Response:

Thank you for your suggestions. According to your requirements, we provided the original uncropped and unadjusted images underlying all blots which could be found in Supporting Information files and named as S2 raw images (S2 Fig. Full western blots supporting Fig 2A-B and S1 Fig A). We also noted that these blots in Supporting Information in our cover letter.

3).We note that you have indicated that data from this study are available upon request. PLOS only allows data to be available upon request if there are legal or ethical restrictions on sharing data publicly.

We note that you have stated that you will provide repository information for your data at acceptance. Should your manuscript be accepted for publication, we will hold it until you provide the relevant accession numbers or DOIs necessary to access your data. If you wish to make changes to your Data Availability statement, please describe these changes in your cover letter and we will update your Data Availability statement to reflect the information you provide

Author Response:

Thank you for your comments. After careful consideration, we decided to make changes to our Data Availability statement. All data are fully available without restrictions, and All relevant data are within the manuscript and its Supporting Information files. Thanks again.

**Comments of Reviewer #1**

1). My main doubts relate to the analysis of TYMS in the context of cell proliferation. First, since the authors decided to analyze the panel of ESCC lines, why was only the mRNA level shown and not the protein level? As it is well known, both indicators do not have to correlate, and from the point of view of the thesis that the authors want to prove, the actual level of TYMS in cells is more important. In addition, in order to prove their thesis, the authors should analyze the proliferation rate of more cell lines and analyze whether the increase in proliferation correlates with the level of TYMS protein in these cells.

Author Response:

Thanks a lot for your suggestion and comments. Indeed, there is no necessary connection between mRNA and protein, and it is necessary to detect the levels of TYMS protein in Multiple esophageal squamous cell carcinoma cell lines. TYMS protein expression in ESCC cell lines was detected through Western blot (WB), and Related results could be found in Figure 2A. According to the results of WB and Q-PCR, both te-5 and te-14 with low TYMS were firstly used for the overexpression of TYMS. However, we failed to construct the stable transgenic cell line with TYMS overexpression in te-5 and te-14, and other cell lines had to used for the constructing. Finally, constructing of TYMS overexpression was successed in TYMS150 and 180, and both cell lines were used for the future experiments (such as MTT, clone formation assay, and so on). Thanks again.

2). Secondly, the authors' results showing different prognoses for patients with high TYMS levels depending on the treatments performed after surgery are very interesting but may confuse readers. Therefore, the authors should try to explain this phenomenon, especially in the context of other works, such as 10.3892/ol.2010.227, and other types of cancer in which a high level of TYMS is observed.

Author Response:

Thanks a lot for your comments. The study (10.3892/ol.2010.227) provided from you is necessary to prove our view. The patients with low TYMS expression exhibited a worse prognosis from GEPIA online database (Fig 1B), and the same results could be found in survival analysis of ESCC patients with radiotherapy and chemotherapy (Fig 1E). However, Kimura et al. [22] found that ESCC patients with high TYMS mRNA expression levels had a significantly shorter survival after surgery, and none of the patients received chemotherapy or radiation therapy prior to or following surgery. In present study, the survival analysis in ESCC patients without radiotherapy and chemotherapy after surgery showed similar results (Fig 1D). The large prospective analysis from Niedzwiecki et al.[23] showed that the high tumor TYMS levels were associated with improved overall survival (OS) and disease-free survival (DFS) following adjuvant therapy for colon cancer. The non-small cell lung cancer (NSCLC) patients with low TYMS expression also had statistically significantly longer OS and progression-free survival (PFS) than those with high TYMS, and low TYMS protein expression is a favorable predictive factor for better OS/PFS in NSCLC patients [24]. Collectively, this study demonstrates that the TYMS level in tumor tissues may be a useful marker to predict the postoperative OS of cancer patients, and the use of anti-tumor drugs (such as 5-fluorouracil) could significantly improve the prognosis of ESCC patients with high TYMS expression. These could be found in revised manuscript in page 11. Thanks again for your help.

3). The discussion of the results should be much more detailed. As a rule, each result should be discussed, and each Figure should be cited in the text (no citation for Fig. 1C, D, and E; only general citation for Figs. 3 and 4).

Author Response:

Thanks a lot for your comments. Your suggestions are absolutely right. According to your suggestion, we checked the paper, and also cited all figure in correct position.

Discussions related to the results were also supplemented, which could be found in the Results and Discussion of revised manuscript. Thanks again for your suggestions.

4). In addition, since the authors are analyzing the role of TYMS in cancer progression, other recent papers related to this topic should also be discussed (colon cancer, breast and lung cancer). Moreover, data reported by Kimura et al. about TYMS dehydrogenase mRNA levels in esophageal cancer should be interpreted.

Author Response:

Thanks very much for your comments. The role of TYMS in cancer progression was discussed, and some related study also were cited. The detailed content is as follows: The patients with low TYMS expression exhibited a worse prognosis from GEPIA online database (Fig 1B), and the same results could be found in survival analysis of ESCC patients with radiotherapy and chemotherapy (Fig 1E). However, Kimura et al. [22] found that ESCC patients with high TYMS mRNA expression levels had a significantly shorter survival after surgery, and none of the patients received chemotherapy or radiation therapy prior to or following surgery. In present study, the survival analysis in ESCC patients without radiotherapy and chemotherapy after surgery showed similar results (Fig 1D). The large prospective analysis from Niedzwiecki et al.[23] showed that the high tumor TYMS levels were associated with improved overall survival (OS) and disease-free survival (DFS) following adjuvant therapy for colon cancer. The non-small cell lung cancer (NSCLC) patients with low TYMS expression also had statistically significantly longer OS and progression-free survival (PFS) than those with high TYMS, and low TYMS protein expression is a favorable predictive factor for better OS/PFS in NSCLC patients [24]. Collectively, this study demonstrates that the TYMS level in tumor tissues may be a useful marker to predict the postoperative OS of cancer patients, and the use of anti-tumor drugs (such as 5-fluorouracil) could significantly improve the prognosis of ESCC patients with high TYMS expression. Thanks again for your suggestion.

5). Provide detailed information about statistical analysis in Figure legends.

Author Response:

Thanks very much for your comments. According to your suggestions, information about statistical analysis was modified and as follows: Data were presented with mean ± standard deviation (S.D.) from least three independent experiments. All statistical analysis was performed using SPSS software (version 17.0, SPSS Inc., Chicago, IL, USA), and data graphs were generated using GraphPad Prism 5 Software (GraphPad Software Inc. La Jolla, CA, USA). Survival curves were constructed using the Kapla-Meier method and differences in survival were evaluated using the log-rank test. Data homogeneity of variance and normality were tested using the Levene tests and Kolmogorov–Smirnov, respectively. *p* values were performed using Student’s t-test and one-way ANOVA analysis between groups, and a value of *p* < 0.05 was considered as statistically significant which was denoted with *. Besides, more detailed information about statistical analysis was provided in Figure legends, which could be found in revised manuscript. Thanks again.

6). In lines 243-244, the Authors stated that "[…] cell lines with lower TYMS mRNA were chosen for TYMS over-expression experiment". However, KYSE180 cells revealed a relatively high TYMS mRNA level. Why did the authors choose this cell line, not those with much lower TYMS expression, such as TE-9 - TE-15?

Author Response:

Thanks very much for your comments. In present study, TYMS protein expression in ESCC cell lines was detected through qRT-PCR and Western blot (WB), and Related results could be found in Figure 2 A. According to the results of WB and qRT-PCR, both te-5 and te-14 with low TYMS were firstly used for the overexpression of TYMS. However, we failed to construct the stable transgenic cell line with TYMS overexpression in te-5 and te-14, and other cell lines had to used for the constructing. Finally, the stable TYMS expression cell line was constructed successfully in TYMS150 and 180, and the efficiency in TYMS over-expression cells were also confirmed by qRT-PCR and Western blot (Fig 2B). Thanks again.

7). In order to confirm the hypothesis of the role of TYMS in the regulation of proliferation via the NRF-2-ROS pathway, it is necessary to silence TYMS in cells with high protein levels (for example, HET-1A or TE-1) and show that the proliferation and oxidative potential of the cell decreases (level genes, ROS level, etc.).

Author Response:

Thanks very much for your comments. Your viewpoint is correct, and it is necessary to silence TYMS. In the early stages of this study, we tried to establish a TYMS stable knockout cell lines in TE-1 and TYMS180 using Lentivirus Vector. It is hard to construct TYMS-silencing cells, and we fail until the paper submitted to the journal. Fortunately, we finally succeed to construct TYMS-silencing cells, and the related rsults could be found in Figure S1. Thanks again.

8). More detailed information on the methodology should be given. E.g., I couldn't find any detailed information about the procedure for stripping and re-probing a membrane. What was the average quality of the RNA extracted (A230/280 ratio)? Why was only one housekeeping gene selected? Has any analysis been performed based on any software (for example geNorm or others)? Please provide the sequence of the primers used (The Table 1 is missing).

Author Response:

Thanks very much for your comments. According to your suggestion, more detailed information about qRT-PCR analysis was provided, and as follows: Total RNA was extracted from 5.0 x 10^5^ cells using the RNA extraction kit according to the manufacturer's instructions. The concentration of isolated RNA extracted were quantified at 230nm, 260nm, and 280nm using NanoDrop Spectrophotomete (Thermo Scientific, USA), and only those samples with a 260nm /280 nm ratio between 1.8-2.1 and a 260nm /230 nm ratio of more than 2.0 were used for further analysis. Using MMLV reverse transcriptase (Takara Shuzo Co. Ltd., Kyoto, Japan), the first-strand cDNA was synthesized from the 200 ng of isolated total RNA. Then, the expression of gene mRNA was determined using the SYBR® Green PCR Master Mix (Vazyme Biotech Co., Ltd) by ABI 7500 Real-Time PCR System (Applied Biosystems, Foster City, CA, USA). The qRT-PCR specific primers were designed based on genes sequences from the NCBI GenBank database using Primer software (Premier Biosoft, Palo Alto, CA, USA), and were shown in Table 1. The qRT-PCR reaction mixture was shown below: 0.5 μL each forward and reverse primers (10 μM), 1 μL cDNA template, 10 μL Realtime PCR Super mix (2×), and RNase free dH_2_O to adjust to 20 μL. Using the 2^-ΔΔCt^ method, the relative quantification of genes mRNA expression was achieved by concurrent amplification of the GAPDH endogenous control. These could be found in page 7.

9). Calling clones of TYMS overexpressing cell lines WT may be misleading as this designates Wild Type. In order to avoid confusing the readers, I suggest changing it, e.g., KYSE-Tyms.

Author Response:

Thanks very much for your comments. Indeed, the word “WT ” might be misleading, and all “KYSE-WT” in figures or paper was replaced with TYMS in revised manuscript.

10).ML385 treatment. First, it is an NRF-2 inhibitor, not a TYMS, as the authors presented. Secondly, what was its concentration, because rather not 20 mg/mL as indicated in the manuscript (10 mM corresponds to 5.11 mg/mL). Why did the authors decide to incubate the cells with the inhibitor for such a long time (96h)?

Author Response:

Thanks very much for your comments. We are so sorry for the mistakes due to our careless. After confirmation, the stock solutions of ML385 were 5mM, and cells were treated with 5.0µM ML385. After that the cells were added with ML385, the significant difference was not observed in 24h or 36h. After a series of pre tests, 72 h rather than 96h was chosen as the action time for the further experiments. We apologize again for our carelessness．Thanks again for your help.

11). It is known that the MTT test is based on reducing a tetrazolium salt to formazan crystals. It is a process that is sensitive to oxidative changes that may affect the results obtained. If so, why did the Authors choose MTT when studying the TYMS-NRF-2-ROS pathway and not a more indifferent test like neutral red uptake or SRB?

Author Response:

Thanks very much for your comments. Your suggestion is very professional. As you know, MTT assay is a routine method for detecting cell proliferation, and we overlooked this point when designing experimental plans. There might be some effect of changed ROS on MTT test, and we try to minimize these impacts as much as possible (such as control group and experimental group was added with an equal amount of excessive MTT). Your suggestion is very correct, and We will take your suggestion into consideration in further experiment. Thanks again.

12). Figure 4- Figure A is ESCA instead of ESCC. Figure B please provide a better quality of images. Figure D and E there must be a mistake; survival time ends at 1200 months (100 years).

Author Response:

Thanks a lot for your comments. After a careful check, we are very sorry that the ESCA was mistakenly written as ESCC, which had been corrected. Figure B was replaced with a better quality of images. The mistake in Figure D and E has been revised, and the day (survival time) was mistakenly written with months.

13) . Additionally, please provide the statistical analysis for proliferation curves.

Author Response:

Thanks a lot for your comments. The statistical analysis for proliferation curves was provided in Statistics analysis of revised manuscript, and as follows: *p* values were performed using Student’s t-test between groups, and a value of *p* < 0.05 was considered as statistically significant which was denoted with *. The significant difference had bee marked with * in all proliferation curves.

**Comments of Reviewer #2**

1) . In the “Abstract” and the first subheading "TYMS expression and prognosis analysis of esophageal cancer ", the authors mentioned “TMSB10”, which encodes thymosin beta 10, in their study. However, the reviewer was not clear and confusing what is its relevance to this study.

Author Response:

Thanks a lot for your comments. We are very sorry for the mistakes. Due to our negligence, the word “TYMS” was mistakenly written as “TMSB10”. After careful check, the mistakes had been corrected in revised manuscript.

2) . Figure 1— Although the authors indicated that the survival analysis in 58 ESCC patients with radiotherapy and chemotherapy after surgery showed opposite results to the survival analysis in 97 ESCC patients without radiotherapy and chemotherapy after surgery (Fig. 1D, 1E), there was not any explanation for this discrepancy. Moreover, there was no explanation for the correlation between high TYMS expression and a better patient prognosis shown in Fig. 1B, which actually conflicts to the role of TYMS in ESCC carcinogenesis. The acronyms “ESCA” and “ESCC” should be spelled out in the figure legend.

Author Response:

Thanks a lot for your comments. Firstly, the acronyms“ESCA” and “ESCC” was spelled out in the figure legend. Secondly, there was indeed not any explanation for above discrepancy, and more detail information could be found in modified manuscript, and was as follows: The patients with low TYMS expression exhibited a worse prognosis from GEPIA online database (Fig 1B), and the same results could be found in survival analysis of ESCC patients with radiotherapy and chemotherapy (Fig 1E). However, Kimura et al. [22] found that ESCC patients with high TYMS mRNA expression levels had a significantly shorter survival after surgery, and none of the patients received chemotherapy or radiation therapy prior to or following surgery. In present study, the survival analysis in ESCC patients without radiotherapy and chemotherapy after surgery showed similar results (Fig 1D). The large prospective analysis from Niedzwiecki et al.[23] showed that the high tumor TYMS levels were associated with improved overall survival (OS) and disease-free survival (DFS) following adjuvant therapy for colon cancer. The non-small cell lung cancer (NSCLC) patients with low TYMS expression also had statistically significantly longer OS and progression-free survival (PFS) than those with high TYMS, and low TYMS protein expression is a favorable predictive factor for better OS/PFS in NSCLC patients [24]. Collectively, this study demonstrates that the TYMS level in tumor tissues may be a useful marker to predict the postoperative OS of cancer patients, and the use of anti-tumor drugs (such as 5-fluorouracil) could significantly improve the prognosis of ESCC patients with high TYMS expression.

3) . Figure 2— As TYMS may be highly expressed in ESCC cell lines, it would be better to select a high TYMS-expressing ESCC cell line and knockdown of TYMS in this cell line for a comparative investigation. It is not clear why the authors chose KYSE180 for TYMS-overexpression experiments as this ESCC cell line express relatively higher levels of TYMS compared to the other ESCC cell lines (Fig. 2A, 2C).

Author Response:

Thanks a lot for your comments. In the original paper, the results of mRNA expression by QRT-PCR was showed in Multiple esophageal squamous cell carcinoma cell lines, and TYMS protein expression in ESCC cell lines was further detected through Western blot (WB). The Related results could be found in Figure 2 A. According to the results of WB and Q-PCR, both te-5 and te-14 with low TYMS were firstly used for the overexpression of TYMS. However, we failed to construct the stable transgenic cell line with TYMS overexpression in te-5 and te-14, and other cell lines had to used for the constructing. Finally, constructing of TYMS overexpression was successed in TYMS150 and 180, and both cell lines were used for the future experiments (such as MTT, clone formation assay, and so on). Moreover, it is necessary to silence TYMS as you As you pointed out. In the early stages of this study, we tried to establish a TYMS stable knockout cell lines in TE-1 and TYMS180 using Lentivirus Vector. It is hard to construct TYMS-silencing cells, and we fail until the paper submitted to the journal. Fortunately, we finally succeed to construct TYMS-silencing cells, and the related rsults could be found in Figure S1. Thanks again.

4) Figure 3- It is still unclear why overexpression of TYMS causes upregulation of NFE2L2 in ESCC cells. The reviewer wonders if knockdown of TYMS could downregulate NFE2L2 expression in ESCC cell lines. Furthermore, the NFE2L2-knockdown experiments were missing for confirming the effects of NFE2L2 upregulation in TYMS-induced ESCC cell proliferation.

Author Response:

Thanks a lot for your comments. Your suggestion is very necessary for the present study to clear the mechanism of overexpressed TYMS on Nrf2. As we mentioned in earlier response, we tried to establish a TYMS stable knockout cell lines in TE-1 and TYMS180 using Lentivirus Vector in the early stages of this study. However, it was hard to construct TYMS-silencing cells, and we fail until the paper submitted to the journal. Fortunately, we finally succeed to construct TYMS-silencing cells, and also added relevant experiments in TYMS-silencing cells. The related rsults could be found in Figure S1. The Nrf2-knockdown experiments were also difficult. ML385, an NRF-2 inhibitor, was used to replace the Nrf2-knockdown experiments, and confirmed that ML385 treatment in TYMS-overexpression cells re-increased the oxidative stress levels and the slowing-down cancer cell proliferation. Thanks again.

5) . Figures 2D, 2E, 4A, 4B — there was no statistical comparison between control and experiment groups.

Author Response:

Thanks a lot for your comments. The statistical comparison between control and experiment groups in Figure 2D, 2E, 4A, 4B was carried out, and the a value of *p* < 0.05 was considered as statistically significant which was denoted with *.

6). It would be more interesting and impactful to study the mechanisms underlying the depletion of TYMS mRNA in high TYMS-expressing ESCC cell lines, such as to examine the gene expression of NRF2 and NRF2-dependent antioxidant enzymes and their functional regulation in ROS homeostasis and ESCC cell growth in cell-based and animal models.

Author Response:

Thanks a lot for your comments. Your suggestion is very professional and meaningful. The successful construction of TYMS-silencing cells ensured the smooth progress of the experiment which partly confirmed the hypothesis of the role of TYMS in the regulation of proliferation via the Nrf2-ROS pathway. Thanks again.

Following the comments of reviewers, the manuscript has been revised. We hope that the revised manuscript and the accompanying responses will be sufficient to make it suitable for publication in journal of Plos One. Your kind consideration will be highly appreciated.

With best wishes.

Jian Yang
