# Supplementary material for: Thymidylate synthase promotes esophageal squamous cell carcinoma growth by relieving oxidative stress through activating nuclear factor erythroid 2-related factor 2 expression
Source: PLoS One. 2023 Sep 8;18(9):e0290264. doi: 10.1371/journal.pone.0290264 (PMC10490860; doi:10.1371/journal.pone.0290264)
Supplement: Attachment — Submitted filename: Responses to Reviewers.doc [file pone.0290264.s005.doc]

**Responses to Reviewers**

Dear Editor

We want to thank you for your useful comments and suggestions on our manuscript. We have revised the manuscript accordingly, and detailed corrections are listed below:

**Journal Requirements:**

Please review your reference list to ensure that it is complete and correct. If you have cited papers that have been retracted, please include the rationale for doing so in the manuscript text, or remove these references and replace them with relevant current references. Any changes to the reference list should be mentioned in the rebuttal letter that accompanies your revised manuscript. If you need to cite a retracted article, indicate the article’s retracted status in the References list and also include a citation and full reference for the retraction notice.

Author Response:

Thank you for your suggestions. We have reviewed the reference, and ensure that it is complete, correct, and meet PLOS ONE's style requirements.

**Comments of Reviewer**

1). This part is still a big confusion to the reviewer and maybe to the readers as well. Although the authors found out other reports to support their findings “TYMS promotes ESCC cell growth by relieving oxidative stress through activating Nrf2 expression” (e.g., Kimura et al. ESCC patients’ outcome [ref. 22], NSCLC report [ref.24]), the discrepancy in the relationship between the expression levels of TYMS and the outcome of cancer patients receiving chemotherapy/radiotherapy or not remains to be not addressed or discussed (Figure 1). Actually, although TYMS is highly expressed in ESCC tissue samples in the GEPIA data, the patients with high TYMS expression exhibit a better prognosis (Figures 1A and 1B). The similar trend can be found in Figure 1E, while these patients have received radiotherapy and chemotherapy. In contrast, the survival curve in patients without radiotherapy and chemotherapy indicates that the patients with high TYMS expression exhibit a worse prognosis (Figure 1D), which favors to the authors’ present study and experimental setting, that is “TYMS overexpression promotes ESCC cell growth in the condition without chemotherapy and/or radiotherapy”.
Author Response:

Thanks a lot for your comments. We are so sorry for the confusion. Indeed, the complex results of survival analysis greatly confuse the reviewer and readers. The survival analysis results were easily disturbed by multiple factors (such as chemoradiotherapy). In Ref.22 and 24, none of ESCC and NSCLC patients included in the analysis received chemotherapy or radiation therapy prior to or following surgery, and results based on this were reliable: High TYMS mRNA expression levels had a significantly shorter survival after surgery. Ref.23 also confirmed the above point, and high tumor TYMS levels were associated with improved OS and disease-free survival (DFS) following an adjuvant therapy for colon cancer. However, we often overlook this point, and may get a contradictory result. After careful consideration, we decided to delete the survival analysis results to avoid this confusion from complex results, and only Figure 1A was reserved. The deleted results done not affect the conclusion of present study, and the viewpoint (ESCC patients with high TYMS expression exhibit a worse prognosis) was also supported by Ref.22. Thanks again for your comments and suggestion.
2). The authors have to clearly distinguish the clinical sample data with or without chemotherapy/radiotherapy because these two conditions seem to lead to opposite patients’ outcome. The current description for Figure 1 and the second paragraph in the discussion section kind of mix up these two conditions (i.e. with or without chemotherapy/radiotherapy) which is confusing and does not provide any more new insights.

Author Response:

Thanks a lot for your comments. I am so sorry again for the confusion from the survival analysis results. The detailed explanation could be found in Author Response 1. Thanks again for your suggestion.

3). Since chemotherapy and radiotherapy cause an increase in intracellular ROS that can lead to apoptosis via extrinsic or intrinsic pathways and based on the authors’ present study that TYMS activates Nrf2 expression and the following ROS scavenger events, it would be better to discuss the possibility why high TYMS expressing patients receiving chemotherapy and radiotherapy may have a better clinical outcome. Instead, the current discussion for the reports of the clinical outcome of patients who receive chemotherapy and radiotherapy or not would be better to move to the description for Figure 1 and mention that it seems there are two clinical conditions for the roles of TYMS in patients' survival and the author will focus on studying the condition without chemotherapy and radiotherapy.
Author Response:

Thanks a lot for your suggestions. The present study did showed that the high TYMS expressing patients receiving chemotherapy and radiotherapy may have a better clinical outcome. After a large number of investigation on the literatures, we still unable to reasonably explain this result, and it may be resulted from multiple factors. Therefore, it is a interesting clinical topics, and will be our future research focus. We also hope to expand the sample size verification to further confirm the relationship between the TYMS expression and radiotherapy, chemotherapy, prognosis to provide theoretical basis and molecular basis of TYMS for clinical prognostic indicators and drug use. Thanks again for your comments.

4). Are the patients in the GEPIA data receiving chemotherapy/radiotherapy or not and why their data more support that TYMS is an indicator of poor prognosis in ESCC patients?
Author Response:

Thanks for your comments. We do not get the accurate clinical information of patients who chemotherapy/radiotherapy or not. So the results of survival analysis from GEPIA was unreliable, and the results were finally deleted. Thanks again.

5).The authors indicate the results in Figures 1C-E as an unpublished parallel multigroup study (line 272). Are these results their own data? If so, please provide more information in the methods section and it would be better to indicate these are their data because “an unpublished parallel multigroup study” is not a precise description.
Author Response:

Thanks a lot for your comments. These results in Figures 1C-E was from unpublished parallel multigroup study (Transcriptome Sequencing), and the relevant results could not published or be found in any database. We also could not provide more information due to data confidentiality reasons. Therefore, we decided to delete these results. It could not affect the conclusion of present study, and the viewpoint (ESCC patients with high TYMS expression) was also supported by Ref.22 and GEPIA data. Thanks again for your suggestions.

6). The background and discussion sections are poorly written and lack focus.
Author Response:

Thanks for your comments. We have revised the background and discussion sections, and detailed corrections could be found in new manuscript.
7). There are many grammatical and typographical errors throughout the manuscript. For example, line 131 form ->from; lines 288, 292 knockout ->should be knockdown.

Author Response:

Thanks for your suggestion. We are so sorry for the grammatical and typographical errors. We have revised whole paper, and carefully modified these errors. Thanks again for your helps.
